# Gender-based violence experiences among Palestinian women during the COVID-19 pandemic: mental health professionals’ perceptions and concerns

**DOI:** 10.1186/s13031-022-00444-2

**Published:** 2022-04-04

**Authors:** Fayez Mahamid, Guido Veronese, Dana Bdier

**Affiliations:** 1 Psychology and Counseling Department, An-Najah National University, Nablus, Palestine; 2 University of Milano-Bicocca, Milan, Italy

**Keywords:** Gender-based violence, Palestinian women, COVID-19 pandemic, Mental health professionals

## Abstract

**Background:**

In a geopolitically at-risk environment, such as Palestine, gender-based violence (GBV) is still a crucial problem rooted in discriminatory laws and traditional habits exacerbated by the ongoing Israeli military occupation. Moreover, the lack of updated data makes it difficult to grasp the magnitude of the phenomenon entirely; the purpose of the current study was to explore mental health professionals’ perceptions and concerns on GBV among Palestinian women during the COVID-19 pandemic.

**Methods:**

Participants in the study were 30 Mental Health Professionals (MHP) selected using convenience and snowball sampling techniques from among MHP in northern West Bank, Palestine.

**Results:**

A thematic content analysis revealed seven main themes of GBV during the pandemic. Palestinian MHP reported that the increased number of GBV cases among women during the COVID-19, quarantine, physical distancing measures, and closure of non-essential services significantly heightened the risks of GBV among Palestinian women. Moreover, Palestinian women involved with or married to older men or married at a very young age were at risk of GBV more than others. Results of qualitative analysis also showed that Israeli occupation and the political violence characterizing the area for decades (including restriction of movement, house demolitions, separation of family members, etc.) have also exacerbated and increased GBV in the occupied Palestinian territories.

**Conclusions:**

Improving intervention skills and supervision services among Palestinian MHP to help women who face GBV is recommended. Moreover, additional research should be conducted to explore the risk and potential factors of GBV, agency, and coping strategies to deal with GBV.

## Background

A novel coronavirus that was termed COVID-19 by the World Health Organization (WHO) is a “pneumonia of unknown origin” responsible for a worldwide outbreak [30]. COVID-19 is highly contagious and rapidly spreading [55]. At the time of writing (November 23, 2021), there were 256,480,022 confirmed cases of COVID-19, including 5,145,002 deaths, globally [57]. There were 429,333 confirmed cases, with 4524 deaths documented in the occupied Palestinian territory [58].

According to the behavioural immune system (BIS) theory, during epidemics, people are more likely to develop negative emotions (e.g., aversion, anxiety, etc.) and negative cognitive assessment toward self-protection. Furthermore, according to stress and perceived risk theories, public health emergencies trigger increased negative emotions that affect cognitive assessment [28]. For example, fear and stress of the COVID-19 pandemic were associated with several psychological impairments such as depression, anxiety, stress [31, 32, 55].

Moreover, gender-based violence (GBV) rates increased during the COVID-19 pandemic [39]. GBV is best defined as any act that results in physical, sexual or psychological harm or attack against women, including threats of such acts, coercion, or arbitrary deprivation of liberty, whether occurring in public or private life [10].

GBV is considered as the outcome of unequal and unjust social conditions, with gender relations intersecting with other dimensions like race, class, culture, and the economy, as expressed through the concept of structural violence [43]. According to a technical note projecting the impacts of COVID-19 on GBV released by the United Nations Population Fund [49], it was expected that a 6-month lockdown would contribute to a global increase in 31 million cases of intimate partner violence (IPV). Furthermore, service provision disruptions and waves of poverty would result in 13 million child marriages by 2030 [27].

Globally, during the COVID-19 pandemic, GBV and domestic violence increased significantly in the United States. Moreover, domestic violence has increased to 40–50% in Brazil, 30% in France and 18% in Spain [13, 15]. Though, in the Arab world, it was noted that GBV rates increased during the COVID-19 pandemic in many Arabic countries such as Lebanon, Syria, Jordan, and Iraq [2, 18, 33].

In the Palestinian context, it was found that 37% of women were exposed to violence; 58.6% of the women were exposed to psychological violence at least once; while 23.5% were exposed to physical violence; 11.8% to sexual violence; 54.8% to social violence; and 55.1% to economic violence [10].

Accordingly, the current study will explore the GBV from the perspectives of mental health professionals in a Palestinian context characterized by high levels of traumatic stress. Palestinian women are expected to be more vulnerable to GBV and its negative impact on mental health during the COVID-19 outbreak. These women live in a militarily occupied country that exposes the civil population to continuous social, economic, and political burdens [11], with a shortage of mental health care institutions and services [29, 35].

Throughout COVID-19 pandemic GBV, mainly domestic violence rates increased since people worldwide are forced to stay at home for a long time (i.e. prolonged quarantine) with the abuser. Economic and social stressors such as the slowdown in businesses, possible unemployment, and scarcity of basic provisions caused by the closure increased tensions in the household [34, 44, 48]. The reduction in the victim’s social contact with friends and family due to stay at home enforced policies reduced the possibilities for women to create and/or strengthen social support networks, seek help, and escape the condition of violence [36]. On the other hand, economic discrimination and exclusion from the labour market are considered a type of GBV during COVID-19, as women have been pushed into extreme poverty and become less financially secure as governments’ worldwide implemented widespread social distancing, school closures, and work from home policies [50].

Gender-based violence was associated with several psychological impairments among women during COVID-19. For example, Sediri et al. [46] assessed the effect of the COVID-19-related lockdown on Tunisian women’s mental health and gender-based violence,the results showed that most participants reported a high level of distress symptoms (57.3% had highly severe anxiety and depressive symptoms, and 53.1% had extremely severe stress symptoms). Those harmed during the lockdown were found to have more severe depression, anxiety, and stress symptoms. Moreover, it was found that women experiencing intimate partner violence are at high risk of developing mental health problems during the pandemic [4]. Furthermore, gender-based violence was correlated with depression, anxiety, and stress among Palestinian women from the West Bank [56, 55]. Bdier and Mahamid [10] also found that gender-based violence is positively and significantly associated with depression, anxiety, and stress among Palestinian women.

### The current study

Our study aims to qualitatively explore the perceptions and concerns of mental health professionals about gender-based violence experiences and mental health outcomes among Palestinian women during the COVID-19 pandemic. Because of its familistic and traditional societal structure and organization, in Palestine, women have less power to make decisions and are more vulnerable to being physically and psychologically abused than in other, more equalitarian countries. Likewise, stressful life events, husbands’ controlling behaviour, and marital conflicts were related to different forms of GBV [5]. Also, residing in a militarily occupied country was associated with increased risks for GBV, as experiences of occupation-related events increased IPV. Namely, men showed gender inequitable attitudes, while women disagreed with their husbands more [55].

Lack of data and information about GBV in Palestine during the pandemic makes it urgent to shed light on the severe violations of women’s rights throughout the experience of frontline health workers to understand a phenomenon that poses urgent challenges to the health system in the region. Thus, we set out to answer the following questions: Does the COVID-19 pandemic increase rates of gender-based violence among Palestinian women? What types of gender-based violence did Palestinian women experience during the COVID-19 crisis? How does gender-based violence affect Palestinian women’s mental health during COVID-19? Does the Israeli military occupation contribute to exacerbating the risks of GBV among the Palestinian community?

## Method

### Participants

Participants in the study were 30 Mental Health Professionals (MHP) selected using convenience and snowball sampling techniques from MHP residing in the northern West Bank, Palestine. In terms of their professional profiles, respondents comprised Psychologists (12), Psychiatrists (3), Counsellors (10), and Clinical Social Workers (5). Twenty were women, and 10 were men. Participants were aged between 22 and 45 years (mean age males = 34.12 years, *SD* = 4.06; mean age females = 32.05 years, *SD* = 4.77). Mean years of experience males = 15.20, *SD* = 3.60; Mean years of experience females = 12.78, *SD* = 4.10. The study’s inclusion criteria required participants to be: (1) from MHP who work with Covid-19 patients, (2) Palestinian, and (3) native Arabic speakers.

### Instruments and procedures

The data were obtained via semi-structured interviews with MHP in the northern West Bank during the spread of the Covid-19 pandemic in Palestine. Participants were selected using purposive and snowball techniques from mental health institutions that offer sections for the Covid-19 pandemic and mental health centres that offer mental health services for families and patients of the Covid-19 infected. All interviews were administered by local research assistants recruited at An-Najah National University in Nablus, Palestine. The field research staff had received qualitative research methodology training and conducted the interviews following the research purposes. All participants were native Arabic speakers. The sample was recruited as follows: first, we contacted the representatives of the mental health institutions explaining the study aims, the planned research procedure, the number of MHP we intended to interview, and the selection procedure. Then, convenience and snowball sampling techniques were used to identify professionals in each institution interested in participating in the research and being interviewed.

Participation was voluntary, and participants were free to withdraw from the study at any time. Furthermore, a counsellor (a licensed mental health professional) was available to support any participant who might become distressed during the interview. Besides, all participants were provided with contacts in mental health services to seek help if symptoms arose subsequently to participate in the study. The interviews with mental health professionals were conducted personally at mental health centres in October 2021. The shortest interview lasted about 45 min, while the most extended interview lasted 60 min; however, most interviews were around 50 min. The study was reviewed and approved by the IRB of An-Najah University before data collection was initiated.

### Data analysis

All interviews were audio-recorded and transcribed in Arabic by a mother tongue researcher. Thematic content analysis (TCA; [42]) was applied to the written transcripts to identify the main themes emerging from the transcripts. In addition, a bottom-up, data-driven textual analysis was applied to extract categories from the raw data [51]). Each interview was closely examined to pinpoint concepts and not just statements containing similar words. The analytical process comprised the following steps: (a) The researchers conducted open-ended analysis on the participants’ narratives to identify the main research themes; (b) the themes were coded and organized into structured categories; and (c) the categories were discussed and agreed by five judges [25].

We assessed inter-rater reliability by calculating Cohen’s kappa (0.931) [14]. The kappa statistic indicated 93% agreement with the first author’s original coding and was well over the conventional threshold value for acceptance of 0.80 [38].

## Results

The thematic content analysis of the interview transcripts led to the identification of the seven main themes that we will discuss below: *(1) MHP Perception of GBV during the Covid-19, (2) Nature and causes of GBV during the COVID-19 pandemic, (3) Women’s coping strategies to deal with GBV, (4) Women at risk of GBV, (5) GBV and military occupation, (6) GBV and social stigma and (7) Interventions to deal with GBV.*

### Theme one: MHP perception of GBV during the Covid-19

The population was obliged to live in reduced spaces because of the ‘stay at home’ policies required by the authorities worldwide, so women and girls were at an increased risk of domestic violence, abuse, and other forms of GBV. Also, prolonged quarantine and economic and social stressors caused by the COVID-19 pandemic increased tensions in the household so that violence in the home may worsen [48].

The majority of Palestinian MHP reported the increased number of GBV cases among women during the COVID-19. For example, a psychologist who worked at a mental health institution mentioned: “*During the Covid-19 time, the GBV increased among Palestinian women, we have received many cases who suffered from physical and verbal aggression, as many of them asked for therapeutic interventions*” (32 years, male psychologists, City of Nablus).

Another social worker described the GBV among Palestinian women during the COVID-19 pandemic as a consequence of forced cohabitation and previous lack of marital compassion resulting in intensified forms of violence against women; she expressed:

*During the COVID-19 crisis, the increased interaction between husbands and wives led to a high rate of violence directed against women. The GBV increased significantly during the COVID-19 pandemic, especially among families who suffered from a lack of effective communication before the pandemic* (27 years, female social worker; City of Tubas).

Another counsellor explained the reasons behind the increased degree of GBV throughout the COVID-19 pandemic, she stated: “*The difficult economic situation in Palestine due to occupation and a lack of job opportunities increased psychological and social problems among families which increased violence against women*” (45 years, male counsellor, city of Nablus). Accordingly, deteriorated quality of life among the population due to systematic violence and segregation (see theme below) was identified as a crucial antecedent of GBV during the COVID-19.

### Theme two: nature and causes of GBV during the COVID-19 pandemic

Stay-at-home orders, physical distancing measures, and closure of non-essential services significantly heighten the risks of violence to women and children. Confinement in homes has been identified as a risk given that it contributed to higher rates of unemployment, income loss, increased use of alcohol/drugs, declining mental wellbeing, and housing as well-known triggers of men’s violence against women and girls in Palestine. Perpetrators are using the COVID-19 restrictions and threat of COVID-19 infection, intentionally or otherwise, to restrict women’s movements, gain access to women’s residences, and coerce women into residing with them if they usually reside separately [26].

The interviewees reported different forms of GBV Palestinian women faced during the COVID-19 pandemic. A psychologist who works at one of the family protection centres in the city of Jenin stated: “*During the COVID-19 Pandemic, I worked with many women who suffered from different forms of verbal and emotional violence. These women indicated that men became uneasy and attacked them during the pandemic*” (35 years, female psychologist, city of Jenin).

Another clinical psychologist who worked at a mental health centre indicated, “*We received many women who have been beaten by their husbands and threatened with divorce during the COVID-19 crisis, some of these women showed wounds in their faces and bodies*” (30 years, female psychologist, city of Nablus).

One psychiatrist who works at a clinical mental health centre described the scope of GBV during the pandemic; he said, “*I have worked with many cases who suffered from different types of violence, some of these cases were very difficult, and they tried to harm themselves; therefore they receive medical treatments*” (40 years, male psychiatrist, City of Tulkarm).

Another clinical psychologist mentioned, “*The violence that men are subjected to by Israeli soldiers causes their violence against women at home*” (32 years, female psychologist, City of Jenin).

Another social worker reported, “*The impact of the pandemic in Palestine was great concerning domestic violence, especially since the environment is fraught with violence due to the policies of the Israeli occupation*” (37 years, male clinical social worker, City of Nablus).

### Theme three: women coping to deal with GBV

Women’s response to violence is mainly shaped by the circumstances of the abuse and the assessment of available resistance options. Most battered women try diverse strategies to overcome the violence, such as temporary separations, seeking outside help, physical self-defence, and ending the relationship [45]. It should be noted that ending a relationship does not necessarily reduce a woman’s risk, as some partners become even more violent when women leave or attempt to leave [19]. Furthermore, many environmental and cultural factors may limit women’s available options, including community attitudes towards violence, available environmental resources for battered women, and access to financial resources and social support [10].

Several respondents mentioned different cognitive and behavioural coping strategies that women used to deal with GBV during the pandemic. For example, one psychologist stated:

*Women’s reactions toward GBV varied from one to the other, based on their practices, traditions, and the woman’s proneness to disclose and report her exposure to abuse. I have worked with women who expressed their GBV experiences, others were afraid to report GBV experiences, and they were satisfied with silence* (28 years female, psychologist, city of Nablus).

Another clinical social worker explained women copying to deal with GBV; he mentioned: “*I have worked with women who faced GBV, many of them abused their children, and only a few became aggressive with their husbands or asked for divorce and separation*” (39 years, male; clinical social worker; city of Qalqilia).

Another psychologist described the women’s reactions towards GBV throughout the COVID-19 pandemic. She explained: “*Many women who were subjected to verbal and physical violence surrendered to that, only a few women defended themselves, and very few went to the police and official authorities*” (29 years, female psychologist, city of Nablus).

### Theme four: women at risk of GBV

At the individual level, adolescents are considered uniquely impacted by GBV. Their young age and conflictual relationship adjustment can heighten their risk for physical and emotional GBV [17]. In Palestine, those involved with or married to older men or married at a very young age can face GBV and other dimensions of limited relationship power. Abuse during adolescence increases the risk for subsequent health concerns, including depression, suicidal ideation, chronic inflammation, and can set young women on a trajectory for subsequent abuse [17]. The current crisis of GBV is likely to worsen in the context of COVID-19. Mahamid and Bdier [28] research demonstrated that violence against non-employed and low educated women has increased in Palestine since the outbreak.

Our Participants mentioned different categories of Palestinian women at risk of GBV during the Covid-19 Pandemic. One psychologist mentioned:

*Most of the cases that visited our mental centre and asked for help due to being subjected to GBV were young women who did not continue their education. Many of these women talked about their exposure to various forms of GBV during the COVID-19 pandemic* (28 years, male psychologist, city of Tulkarm).

Another clinical social worker described his experiences with women at risk of GBV during the COVID-19 pandemic. He expressed: “*In general, GBV has increased during the pandemic, but there were women who exposed more than others to this type of violence, among these women were housewives and uneducated women*” (26 years, male clinical social worker, city of Nablus).

Another female counsellor who worked with abused women described her experiences with these women, she said:

*In Palestinian society, a significant number of women are exposed to GBV. I found through my work with these women that they consider this violence as normal behaviour, many of them are not even aware that they have been subjected to gender violence, as they consider the violent behaviour of men and their abuse as expected* (27 years, female counsellor, city of Jenin).

### Theme five: occupation and GBV in Palestine

The Israeli occupation and the political violence characterizing the area for decades (including restriction of movement, house demolitions, separation of family members, etc.) have also exacerbated and increased violence against women in the occupied Palestinian territories (oPts) [20]. Political violence increased the pressure on families that became more vulnerable to multiple stressors, undermining cohesion and reshaping gender or age roles [52]. Israeli military violence has created a difficult economic situation, leading to a high percentage of unemployed and economically resourceless women. It has limited the freedom of movement of Palestinians (including women) demolished dwellings either for lack of building permits or as punitive actions and forced eviction [21]. Girls have been reported to have been harassed by Israeli soldiers on the way to school to the extent that their families prefer not to send them to school [7]. One psychologist reported that “*I worked with a girl who was beaten while returning from her school at one of the checkpoints in the city of Nablus, as a result of that the girl suffered from PTSD symptoms and nightmares for a long period*” (29 years, female psychologist, City of Nablus).

One mental health provider mentioned; *I worked with many girls subjected to violence by soldiers at checkpoints in Nablus. Those girls were beaten and humiliated. They were also asked very aggressively to remove their headscarves (Hijab)*” (31 years, female mental health provider, city of Nablus).

Another female psychologist expressed her experiences in working with women who faced GBV, she mentioned:

*The difficult economic conditions that Palestinian face as a result of occupation (closures policies, demolishing houses, preventing work etc.) all of this has made the economic conditions in the West Bank very difficult, which has increased the rates of family conflicts and GBV* (29 years, male psychologist, city of Nablus).

One psychologist added that “violence *against women in Palestinian society is part of the general societal frustration and hopelessness. It is a result of the occupation policies towards Palestinians*” (30 years, male psychologist, city of Jenin).

Based on her experiences with women who suffer from GBV, a female counsellor who worked at a community mental health institution expressed, “*I worked with many women who faced GBV, most of these women lived in families whose members suffered from political violence and Israeli occupation*” (31 years, female counsellor, city of Tulkarm).

### Theme six: GBV and social stigma

Studies found that stigma exacerbates the adverse effects of violence and is associated with re-victimization and a reduced likelihood of help-seeking [8, 16]. This is partly because a typical response to “internalized stigma” is withdrawal from social support. Women survivors of GBV may withdraw from or fail to demand social support because feelings of shame lead them to believe they do not deserve support or are attempting to hide their condition to prevent status loss and discrimination [9].

Our participants mentioned that the stigma process described by survivors was self-blame or stigmatization even when they could not identify what they had done to “cause” the violence. A psychologist mentioned that one of the women who had been subjected to gender-based violence told her, “*You feel that you are the cause, but when you try to analyze what happened and look at what you did to deserve it, you do not get any answer. Still, you blame yourself*” (30 years, female psychologist, city of Tubas).

Another psychiatrist talked about her experiences working with battered women. She mentioned, “*Many of the women I worked with who experienced domestic violence were trying to deny that they had experienced violence, even though they feel injustice, they rejected the idea of divorce and separation of their husbands because of the societal stigma*” (34 years female psychiatrist, city of Tulkarm).

A female psychologist who worked with women who suffered from GBV expressed, “in our society, women cannot talk freely about GBV. They blame themselves and feel guilty when they talk about family issues to psychologists” (32 years female psychologist, city of Tulkarm).

A clinical social worker indicated, “*Women in our society have a moral obligation towards their families, and they feel stigmatized when they talk about problems with their husbands*” (28 years, male clinical social worker, city of Nablus).

### Theme seven: therapeutic interventions to deal with GBV

In Palestine, interventions with GBV are typically grounded in the cognitive-behavioural approach, feminist and psych-educational approaches, or a combination. Services focus primarily on empowering survivors to stop the violence, make decisions about their relationships, and gain safety [40]. Psychosocial interventions can target multiple measurable goals among women who suffer from GBV, such as reducing PTSD symptoms, increasing assertiveness, and developing positive problem-solving strategies among battered women [41].

Our participants indicated they conducted different interventions to help women who faced GBV during the pandemic. Most of these interventions focused on increasing awareness and reducing the harmful effects of GBV. One clinical social worker indicated, “*During the COVID-19 pandemic, we worked with different women groups in Nablus region, these groups focused on raising awareness and empowerment of women who experienced GBV*” (34 years, female clinical social worker, city of Nablus).

Another counsellor who worked with battered women during the COVID-19 in the Jenin region described her experiences with victims of violence. She indicated, “*My work with battered women in Jenin region focused on improving assertiveness and social skills among these women, as well as helping to protect cases at high risk through the Ministry of Social Development*” (31 years, female counsellor, city of Jenin).

A psychologist described her role in working with battered women during the COVID-19 pandemic; she said: “*Working with battered women during the COVID-19 pandemic was not easy. We conducted psycho-educational interventions targeting battered women to raise awareness and social skills. We also held workshops and open days to raise community awareness on GBV*” (33 years, female psychologist, city of Qalqilia).

Our participants indicated that some women who experienced domestic violence had misconceptions about psychotherapy. One psychiatrist mentioned, “*Some women who have been subjected to domestic violence rejected the idea of receiving psychological treatment because they believe that psychological treatment is only for people with mental problems*” (31 years, female psychiatrist, city of Nablus).

Another counsellor who worked with women who experienced GBV indicated, “*Some women who have been subjected to domestic violence and attended to our mental health centre, they believed that treatment should be through medication only, and they did not believe in psychological treatment*” (28 years, male counsellor, city of Tubas).

Another psychologist said, “*I have worked with women who carried false beliefs on psychotherapy, such as psychotherapy is designed for crazy and mad people, not for me*” (37 years, female psychologist, city of Jenin).

Hence, perceptions and concerns of mental health professionals on GBV in Palestine during the COVID-19 outbreak reflected the difficulties of tackling such an issue in a society where patriarchal powers are still dominant. The following section will discuss those difficulties and contradictions that mental health professionals must confront when dealing with violence against women.

## Discussion

Violence against women in Palestine is enrooted in a patriarchal tradition that oversees different interlocked contextual levels, legal, familial, societal. It does not favour women’s emancipation against subjugating powers, exposing them to GBV and other forms of discrimination or subalternity [7]. Moreover, living under military occupation increases the risk of discrimination and victimization of women [1]. Such difficult contextual conditions have been exacerbated during the pandemic due to the ‘stay at home’ policies that reduced distances between victims and perpetrators, in particular, and exacerbated the oPts isolation and marginalization from the international community overall, creating the conditions for a so-called double pandemic [12]. In fact, our interviewees reported an intersection of determinants of GBV during the COVID-19 outbreak that revealed the syndemic nature of violence against women (VAW) during that time. The lack of economic, educational, community-based resources increased the radicated form of masculine domination against women [37]. On the other hand, health workers are daily confronted with an absolute scarcity of resources in the health sector that contribute to exacerbating the syndemic effects of COVID-19 on VAW and GBV. Accordingly, the emersion and the access of GBV in Palestine remains very scarce because of stigma both about VAW itself and the psychological interventions considered a matter of madness and lunaticity [7, 35].

Our interviewees themselves seemed paradoxically trapped between depoliticized and decontextualized perception of VAW and GBV, which tends to medicalize and psychiatrize a social and cultural issue, and the prejudice that almost exclusively women need education and empowerment to raise awareness on GBV [22, 53]. The impact of violence on women’s mental health resulted from the interviews due to the traumatic incident rather than the intersection of physical, psychological, social and contextual factors with a real risk of pathologization and victimization of women. Very few agentic practices and survival skills emerged, making the psychological and psychiatric intervention ultimately one of the few escaping rooms from violence and discrimination. The role of men, even in the recovery process, emerged as very marginal. Economic constraints, the politics of occupation exacerbated in times of pandemic, seemed the only explanations the mental health providers raised as determinants of the perpetrators’ frustration and violence against women. No mention or marginal considerations about socio-cultural factors that foster patriarchy in Palestine emerged from the Palestinian health workers’ discourse. Both stigma and underestimation within the Palestinian community of GBV are more referred to as the sole emersion of violence and the related women’s shame, rather than structural and institutional forms of gender violence that silence women in the Palestinian society [6, 47]. Understated, it emerged how decades of occupation and colonization, fostering a masculine discourse, intersected a patriarchal and familistic organization of the Palestinian society that silenced the competencies of women in reacting to male subjugation and political violence over the years. COVID-19 made it more visible and challenging to confront, relegating women to, already narrowed by the occupation, domestic spaces exposed to colonial and patriarchal domestic violence [23, 54].

### Limitations of the study

The current study has several limitations that may offer opportunities for future research. First, our study targeted Palestinian mental health professionals (psychologists, psychiatrists, counsellors and clinical social workers) who work at mental health centres in the northen West Bank. Exploring perspectives on GBV with different samples of professionals (family physicians, staff nursing professionals, and police officers) is needed. Secondly, the data was collected during the wave of the Omicron variant in Palestine. Hence, the COVID-19 pandemic heightened GBV and IBV, possibly skewing the results. Therefore, future studies are needed to test GBV and IBV within Palestinian contexts over different periods. Thirdly, our study used thematic content analysis to explore GBV among Palestinian women; more studies using mixed methods design are needed. Finally, our study used convenience and snowball sample techniques to select participants of the study. Selecting representative samples from different regions of Palestine is needed in future studies.

## Conclusion

COVID-19 was an accelerator in exacerbating women’s subjugation and marginalization in the Palestinian context [21]. During the pandemic, the interlocked effects of political and military violence intersecting patriarchal traditions undermined women’s positions within the Palestinian communities, augmenting VAW and GBW risks. From the voices of our interviewees, the health sector in Palestine showed to be unprepared to answer the complex phenomenon of GBV and VAW, resulting in a reductionistic medicalized and psychiatrized response [23]. A community response during the pandemic to fight gender-related discrimination is urgent. However, during the pandemic, it is essential to acknowledge the worsening effects of the military occupation on Palestinian society. Health workers should address community-oriented interventions for a comprehensive education on gender equality during and beyond the pandemic [24]. The sole psychological and psychiatric intervention risks reproducing pathologizing and stigmatizing masculine discourses that tend to marginalize and silence women’s voices; on the contrary, community-based interventions are needed to support women taking control over their health resources to combat entrenched systems of historical political and gender-based violence. Historic institutions in Palestine are known to subjugate women and relegate them to the margins of society, as seen in the COVID-19 pandemic [3].

In conclusion, mental health professionals’ concerns about GBV and VAW call for an immediate lift of the Israeli occupation that suffocates the Palestinian civil society and a profound reform of the patriarchal norms and habits in Palestine as a means to overcome the COVID-19 challenges and move toward a due liberation of the Palestinian women and the whole society under occupation [20].

### Statement positioning

We must acknowledge in this paper the different positioning of the authors. One of them, a non-native white and western researcher, must recognize his privilege and western-informed view on gender relations, gaps and inequalities from the Global North. The other two authors are native Palestinians, a man and a woman, living under occupation and constantly exposed to colonial threats and harassment. The findings of this work are the outcome of an ongoing, inspiring, not always comfortable, open dialogue aimed at decolonizing research and intervention practices in Palestine and the Global South in the coming years.

## Data Availability

The datasets generated during and analyzed during the current study are available from the corresponding author on reasonable request.
